# Aging and public health: the Brazilian Longitudinal Study of Aging (ELSI-Brazil)

**DOI:** 10.11606/S1518-8787.201805200supl2ap

**Published:** 2018-10-25

**Authors:** Maria Fernanda Lima-Costa

**Affiliations:** I Fundação Oswaldo Cruz. Instituto René Rachou. Núcleo de Estudos em Saúde Pública e Envelhecimento. Belo Horizonte, MG, Brasil; II Fundação Oswaldo Cruz. Instituto René Rachou. Programa de Pós-Graduação em Saúde Coletiva. Belo Horizonte, MG, Brasil

Brazil is experiencing one of the fastest rates of population aging worldwide^1^. As Brazil has the fifth largest population in the world, the aging of its population has repercussions that transcend the country’s borders. This demographic shift brings both opportunities and challenges not yet fully understood. Promoting active aging^2^ and strengthening economic and social institutions to ensure financial security and provision of adequate health care^3^ are crucial issues.

Large population-based longitudinal studies on aging have been conducted in various countries of the Americas, Europe, and Asia. These studies, known as the Health and Retirement Family of Studies^4^, investigate the social and biological determinants of aging and the consequences of this demographic change for the individual and for society. Although these studies are independent, to meet the demands and particularities of each country, they seek to adopt a common methodology to allow for international comparisons. The Brazilian Longitudinal Study of Aging (ELSI-Brazil), conducted in a nationally representative sample of people aged 50 years and over, is part of this international network^5^. ELSI is funded by the Brazilian Ministry of Health and has the support of researchers from several Brazilian and foreign academic institutions, as well as from policymakers from the Brazilian Unified Health System (SUS) at its various levels. The manuscripts included in this supplement of the Revista de Saúde Pública are fruit of these important collaborations.

The selection of topics covered in these articles was based on the three pillars of the policy framework for active aging of the World Health Organization, i.e., participation, health, and security^2^. Social security is important at all ages, but its demand is even greater later in life. In the Federal Constitution of Brazil, social security is defined as “initiatives of public authorities and society, aimed at ensuring the rights related to health, welfare, and social assistance”^6^. Therefore, social security has been addressed in several manuscripts whilst health was analyzed in all.

High levels of social inequality are found in the majority of topics investigated, with the poorest or those with lower educational level affected most. Inequalities are observed in physical activity^7^, oral health status^8^, limitations to perform basic activities of daily living^9^, frailty^10^, adequate control of hypertension^11^, underutilization of medications due to financial reasons^12^, source of health care^13^, and capacity to work^14^. Social participation^15^ and the fear of falling^16^ are associated with the urban environment. Perceived quality of life is associated with sociability and emotional and instrumental support^17^. Performance in cognitive function tests is shown to be worse in the Northeast region^18^. Back pain and hypertension play a prominent role in the configuration of multimorbidity^19^. Women are the main source of care for those with functional limitations and, among those who worked, a third stopped working to be caregivers^20^. Some indicators of utilization and quality of health services are better among those who have access to the private health system, who have better socioeconomic conditions^13^. Access to primary care is high by international standards^13^, but many hospitalizations could be prevented by more effective actions at this level of care^21^. Differences in the performance of primary care among users of the SUS are found, with a better performance of the Family Health Strategy compared to the traditional basic units^13^. Incapacity to work^14^, as well as the receipt of pensions^22^ are associated with worse health conditions. However, people who receive pensions are more autonomous and have more financial security^22^.

One of the goals of the Post-2015 Development Agenda, supported by 193 members states of the United Nations, is to ensure healthy lives and promote well-being for all at all ages^23^ ^,^ ^24^. Based on the main findings from ELSI, reported in this special issue, the post-2015 development goals may be jeopardized without investments in social protection/security, education, and health, as well as in overcoming the associated social inequalities. The existing Family Health Strategy program needs to be reinforced and improved to meet the needs of older adults. A social care policy at the national level for those with disability is pressing, given demographic trends pointing to a decrease in the availability of informal (non-paid) care^5^. Finally, the results also highlight the need to consider health in the current discussions around extending working lives and potential changes in the rules for pensions, since health conditions are associated with early retirement and decreased work capacity.
